# Highly Efficient Domain Walls Injection in Perpendicular Magnetic Anisotropy Nanowire

**DOI:** 10.1038/srep24804

**Published:** 2016-04-21

**Authors:** S. F. Zhang, W. L. Gan, J. Kwon, F. L. Luo, G. J. Lim, J. B. Wang, W. S. Lew

**Affiliations:** 1 School of Physical and Mathematical Sciences, Nanyang Technological University, 21 Nanyang Link, Singapore 637371; 2 Key Laboratory for Magnetism and Magnetic Materials of Ministry of Education, Lanzhou University, Lanzhou, 730000, People’s Republic of China

## Abstract

Electrical injection of magnetic domain walls in perpendicular magnetic anisotropy nanowire is crucial for data bit writing in domain wall-based magnetic memory and logic devices. Conventionally, the current pulse required to nucleate a domain wall is approximately ~10^12^ A/m^2^. Here, we demonstrate an energy efficient structure to inject domain walls. Under an applied electric potential, our proposed Π-shaped stripline generates a highly concentrated current distribution. This creates a highly localized magnetic field that quickly initiates the nucleation of a magnetic domain. The formation and motion of the resulting domain walls can then be electrically detected by means of Ta Hall bars across the nanowire. Our measurements show that the Π-shaped stripline can deterministically write a magnetic data bit in 15 ns even with a relatively low current density of 5.34 × 10^11^ A/m^2^. Micromagnetic simulations reveal the evolution of the domain nucleation – first, by the formation of a pair of magnetic bubbles, then followed by their rapid expansion into a single domain. Finally, we also demonstrate experimentally that our injection geometry can perform bit writing using only about 30% of the electrical energy as compared to a conventional injection line.

Magnetic domain wall (DW) based memory technology has shown great potential to be used as a universal memory due to their outstanding performance characteristics^1^,^2^,^3^. In such devices, the non-volatile data bits can be accessed without the use of any mechanical parts. However, the first DW memory demonstrated in in-plane magnetic anisotropy (IMA) material has very large DW widths of about 100 ~ 200 nm which limits its scalability^4^,^5^. In addition, the current-driven DW dynamics is very sensitive to external magnetic fields and suffers from a high intrinsic pinning^6^,^7^,^8^,^9^,^10^,^11^. On the contrary, high perpendicular magnetic anisotropy (PMA) nanowires have smaller domain sizes, narrower DWs and much higher DW thermal stability^12^,^13^,^14^,^15^,^16^,^17^,^18^,^19^,^20^,^21^,^22^. The current-induced DW motion in such systems is dominated by the field-insensitive adiabatic torque which moves the DW by changing its structure between Bloch and Neel walls periodically^14^,^15^,^16^,^17^,^18^. Therefore, the threshold current density required for domain wall propagation is given by the energy difference between the two types of DWs. As compared to an IMA system, the threshold current density for DW motion in a PMA system is lower^19^,^20^,^21^,^22^.

For the realization of DW-based memory devices, it is important to first optimize the injection of a DW into the nanowire. Conventionally, DW injection is achieved by applying an electrical current via a thick and conductive stripline deposited on top of the magnetic nanowire. The Oersted field generated by the conductive stripline is used to change the magnetization direction of the magnetic nanowire, with the magnetization direction depending on the direction of the current applied^23^,^24^,^25^,^26^. To create DWs in PMA materials, the minimum amount of magnetic energy required from the injection line is approximated by the product *KV*, where *K* is the effective magnetic anisotropy energy and *V* is the volume of the magnetic domain^27^. While high *K* materials favor the thermal stability of a PMA device, the emerging problem is that it would then require increasing amounts of energy to perform magnetic bit writing. For the conventional method, the current density required to nucleate a DW is approximately ~10^12 ^A/m^2^ 14,15,16,17,18,23,24,25,26,27,28,29. Reducing the energy consumption by improving the injection line design is therefore a necessity. In this article, we propose a highly angular Π-shaped injection line design and experimentally demonstrate that a DW can be deterministically injected with a current amplitude of about 5.35 × 10^11^ A/m^2^ and pulse duration of 15 ns. Both experimental and simulation results show that our design consumes about 30% of the energy required by conventional injection designs.

## Device structure and experiment details

**Figure 1:**
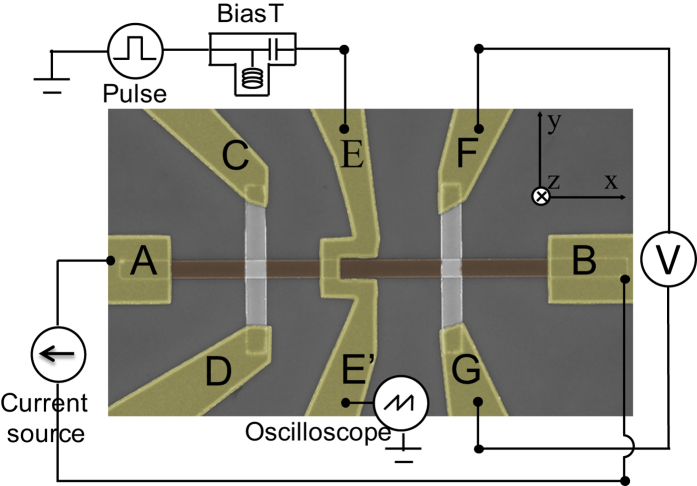
(**a**) False-color scanning electron microscopy image of the device under test, as well as the accompanying electronic test circuit.

Figure 1 shows the scanning electron microscopy image of a fabricated device, consisting of a 350 nm wide PMA nanowire (*AB*), two Ta Hall bars (*CD* and *FG*), the proposed injection line (*EE*’) and electrodes. The Hall bars are used to measure the Hall resistance, *R*_Hall_, via the Anomalous Hall Effect (AHE). The Hall resistivity is empirically fitted by the formula^30^


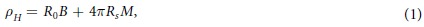


where *B* is the applied magnetic field, *M* is the magnetization per unit volume. *R*_0_ and *R*_s_ are the ordinary and the anomalous Hall coefficient, respectively. For our PMA system, *R*_s_ is substantially larger than *R*_0_. Thus, the *R*_Hall_ is proportional to the perpendicular component of the local magnetization of the nanowire beneath the Hall bar.

**Figure 2:**
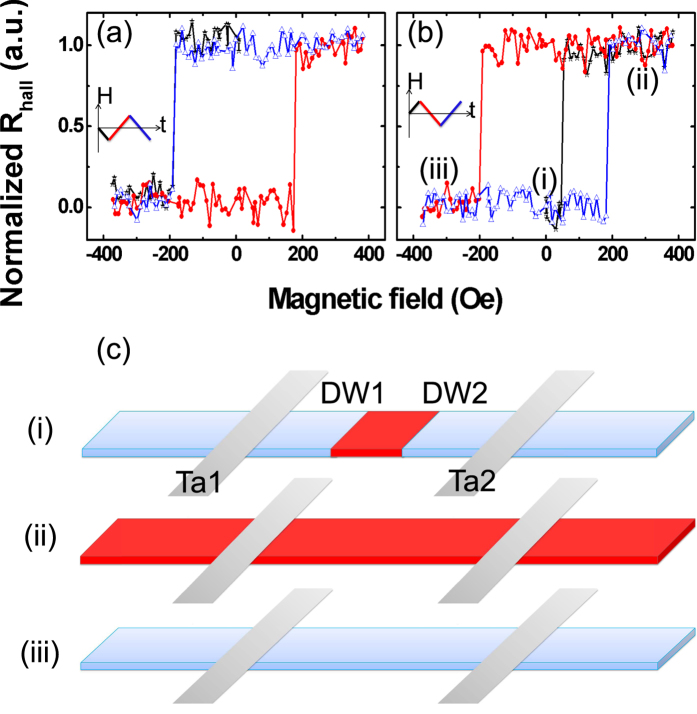
(**a**) Normalized Hall resistance of the PMA nanowire without domain injection, under a 370 Oe sweeping magnetic field. (**b**) Normalized Hall resistance measurement after domain injection, under a 370 Oe sweeping magnetic field. The direction and sequence of the field sweeps are indicated in the figure insets by the line colors. (**c**) Schematic illustrations of the magnetization for (i)–(iii) states denoted in (**b**). The red (blue) color here represents the magnetization direction is up (down).

Figure 2a shows the typical *R*_Hall_ measurement while sweeping a 370 Oe external magnetic field in the *z*-direction, perpendicular to the nanowire. The square loop indicates that the nanowire exhibits a strong PMA. The *y*-axis of the graph is the normalized *R*_Hall_, where 1 and 0 correspond to the complete alignment of the magnetization beneath the Hall bar in the +*z* and −*z* magnetization, respectively. *R*_Hall_ was observed to change at 197 Oe, which corresponds to the magnetization reversal field. The sudden switch can be explained by the nanowire reversal process – a domain first nucleates at a defect in the nanowire and then, by means of DW motion, the domain rapidly expands throughout the nanowire until saturation results. As the threshold field for DW motion is much lesser than the DW nucleation field of the nanowire, only a single change in *R*_Hall_ was expected for each sweep direction.

Before DW injection, a perpendicular field of 370 Oe was applied to saturate the nanowire magnetization in the −*z* direction. DW injection was then carried out by applying a current pulse to the injection line from *E* to *E’* without an external magnetic field. The current pulse generates a local Oersted field with perpendicular field components at the sides of the injection line. If the amplitude and duration (*t*_*p*_) of the pulse reaches the threshold value, a magnetic domain will be introduced in the nanowire as shown in Fig. 2(c)(i), where the red (blue) color means the magnetization direction is up (down). After the domain nucleation process, a magnetic field was swept as shown in Fig. 2(b). As the magnetic field increases from 0–370 Oe, *R*_Hall_ changes sharply from 0–1 at *H* = 57 Oe, which corresponds to the DW propagation field at which the DW is driven across the Hall bar. At 370 Oe, the magnetic nanowire is completely saturated (Fig. 2(c)(ii) and (iii)) and *R*_*Hall*_ only switches at *H* = 197 Oe with the next field sweep.

**Figure 3:**
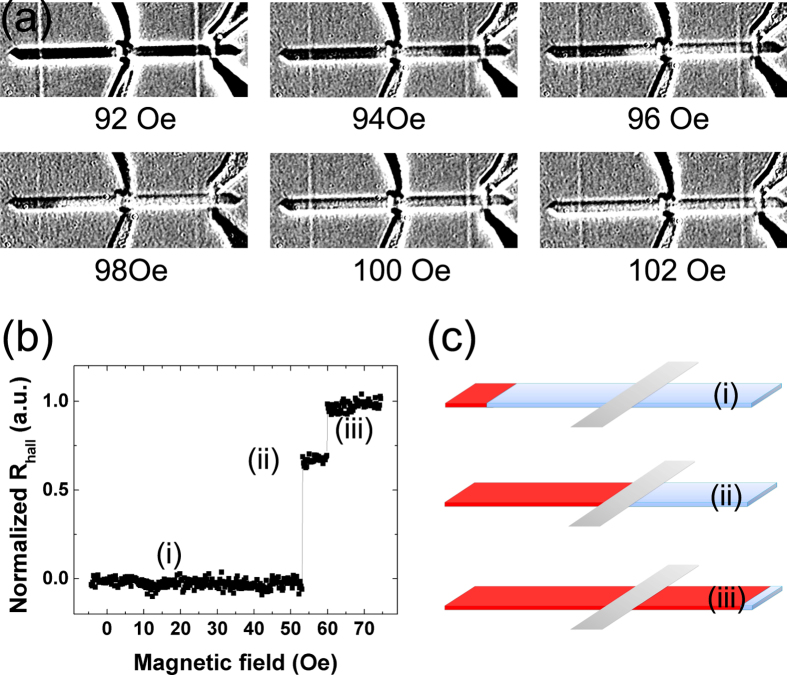
(**a**) Polar Kerr images of a 2 μm wide PMA nanowire after domain injection while sweeping a small magnetic field from 92–102 Oe in 2 Oe steps. An image of the saturated nanowire was then used for background subtraction. The white (black) contrast represents the magnetization direction is up (down). (**b**) Normalized Hall resistance measurement of the 350 nm wide PMA nanowire after domain injection, with magnetic fields weaker than the saturation field. (**c**) Schematic illustrations of the magnetization for (i)–(iii) states denoted in (**b**). Due to the device symmetry, only the right half of the device was shown.

Figure 3a shows the polar Kerr images of a 2 μm wide PMA nanowire after domain injection while sweeping a small magnetic field. To obtain the Kerr images, a nanowire with an injected domain was imaged from 92–102 Oe in 2 Oe steps. An image of the saturated nanowire was then used for background subtraction. The white (black) contrast represents the up (down) magnetization direction. As shown in Fig. 3(a), a magnetic domain was successfully injected into the nanowire underneath the injection line and the domain expands gradually with increasing magnetic field. In a defect-free nanowire, the DWs will keep moving until they reach the end once the threshold propagation field is reached. However, in our experiment, we observed that the DWs are pinned at defects along the nanowire originating from the uneven nanowire sidewalls. The pinning phenomenon was also observed in *R*_Hall_ measurements of the 350 nm wide nanowire performed with magnetic fields weaker than the saturation field as shown in Fig. 3(b). Following the injection of a domain (Fig. 3(c)(i)), the magnetic field was increased from 0–75 Oe. Two distinct *R*_*Hall*_ steps at 53 Oe and 59 Oe were observed. This suggests that the DW has propagated to the Hall bar at 53 Oe (Fig. 3(b)(ii)) and remains pinned until 59 Oe was applied (Fig. 3(b)(iii)). The 6 Oe pinning field may result from the random defects or the interfacial interaction between the nanowire and the heavy metal Ta at the nanowire edge.

### Domain injection probabilities

**Figure 4:**
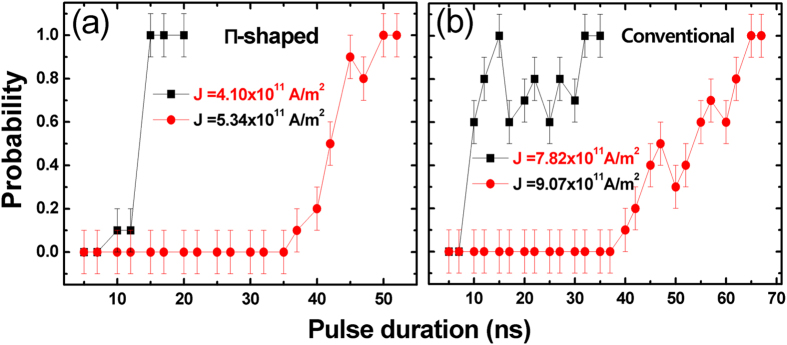
Domain wall injection probabilities as a function of pulse duration and current density for both (**a**) Π-shaped and (**b**) conventional injection line. Each measurement was repeated 10 times.

By measuring the magnetic field at which *R*_Hall_ switches, we were able to determine whether a DW has been injected. Subsequently, 10 measurements for each injection current and pulse duration were performed to obtain the DW injection probability as shown in Fig. 4. Our proposed Π-shaped structure was found to be able to deterministically inject a DW pair in only 15 ns using a low current density of 5.34 × 10^11 ^A/m^2^. Lowering the current density to 4.1 × 10^11 ^A/m^2^ results in an increased threshold *t*_*p*_ of 50 ns. For the conventional method, the current pulse must be larger than 7.82 × 10^11^ A/m^2^ with *t*_*p*_ of 70 ns, or increasing the current density to 9.07 × 10^11^ A/m^2^ results in a decreased *t*_*p*_ of 32 ns. Power consumption, *E*, for magnetic bit injection can be calculated by *E* = *I*^*2*^*Rt*_*p*_, where *I* is the current passing through the injection line and *R* the resistance of the injection line. Thus, our Π-shaped structure is calculated to consume only about 30% of the energy used in conventional method. The relatively low threshold *t*_*p*_ and current density observed in our experiments is attributed to the highly shaped current density and localized magnetic field generated by our proposed injection line.

Under an external magnetic field, the rotational behavior of spins can be modeled by the Landau-Lifshitz-Gilbert equation from where we obtain^31^


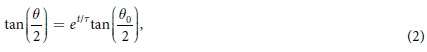


where *θ* and *θ*_*0*_ is the angle between the magnetic moment and the effective field at *t* and at *t* = 0, respectively. 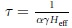 is the spin lattice relaxation time, where *α* is the Gilbert damping parameter, *γ* is the gyromagnetic ratio and *H*_eff_ is the effective magnetic field which is equal to the in-plane saturation field. After a sufficiently long time *t* = 3*τ*, the magnetic moments become almost aligned along the effective field *H*_eff_. Using the measured values of *H*_eff_ = 3000 Oe and the values of *γ* = 2.21 × 10^5^ 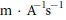 and *α* = 0.02 from literature^22^,^26^, the reversal duration was evaluated to be about 2.8 ns for our device. To account for the significant difference between the calculated value and experimental observations, we further performed micromagnetic simulations.

### Simulations

**Figure 5:**
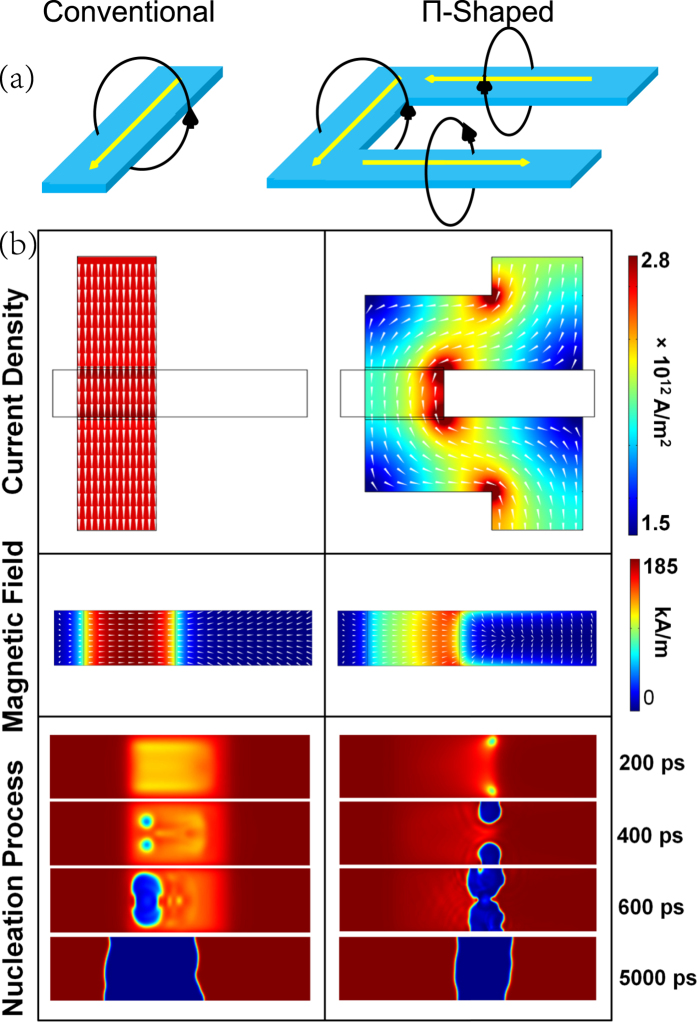
(**a**) Schematic diagram of the current flow and its corresponding magnetic field. (**b**) Comparison of simulated current density distribution of the stripline, magnetic field profile acting on the PMA nanowire and time-resolved magnetization between the conventional (left) and the Π-shaped injection line (right). The current and pulse duration were taken at threshold conditions with values of 220 mA (129 mA) and 935 ps (1350  ps) for the conventional (Π-shaped) injection line. Where applicable, the white cones indicate the in-plane orientation of the vector fields.

Figure 5a shows the schematic diagram of the current flow and its corresponding magnetic field. A significant advantage of Π-shaped stripline is each side of the 3 striplines produces a magnetic field that is oriented in the same direction in the center, producing an effective magnetic field that is stronger than that of a straight stripline. To understand the magnetization reversal processes, COMSOL Multiphysics and mumax^3^ micromagnetics were used to perform simulations. The geometry of the devices-under-test was created in COMSOL to accurately model the spatial distribution of the current density and its resultant Oersted field as shown in Fig. 5(b). The threshold current density for deterministic nucleation (Jth) at 0 K for deterministic nucleation at 0 K was used. Applying the electrical currents with a rise time of 300 ps in mumax^3^, *J*_th_ was determined to be 220 mA and 129 mA, corresponding to an average current density of 2.9 × 10^12 ^A/m^2^ and 1.7 × 10^12^ A/m^2^, respectively. From the simulations, it is clear that the Π-shaped injection line has another two advantages. Firstly, as the Oersted field decays with distance squared, electrical current far away from the nucleation site does not contribute significantly to the Oersted field at the nucleation site. The highly angular design minimizes this type of inefficiency by focusing the current into a narrow region that creates a strong and localized magnetic field at the nucleation site. Secondly, while a threshold magnetic field is required to initiate domain wall nucleation, that magnetic field need not be acting uniformly on the magnetic nanowire. A small localized region of high magnetic field can also initiate the nucleation of a small metastable domain which will easily spread out to form a larger and more stable domain. This is more efficient than applying a uniform magnetic field to nucleate a domain.

On the bottom right side of Fig. 5(b), the time resolved magnetization dynamics of the Π-shaped injection geometry is shown. Upon application of a magnetic field with a 300 ps rise time, spin waves spin wave excitations were observed. After a few oscillations, two metastable magnetic bubble domains are nucleated at the position of the localized magnetic field. The two domains eventually expand in size with the assistance of the Oersted field and forms a complete DW pair after 600 ps. Given sufficient time for the magnetization to relax, it is apparent that the Π-shaped injection geometry produces a narrower domain.

**Figure 6:**
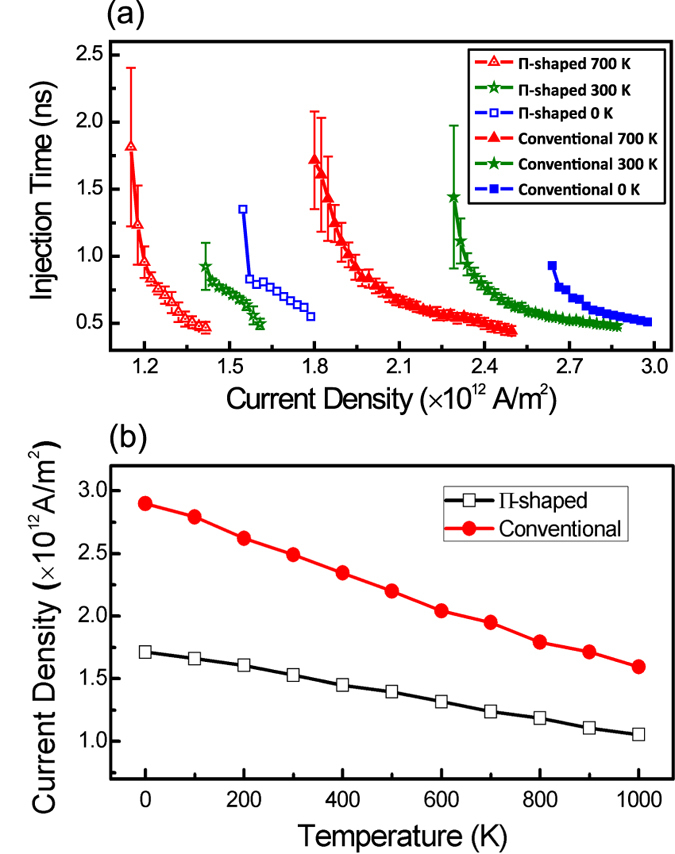
(**a**) Threshold injection pulse duration as a function of current density for both injection lines at different temperatures. The average of 25 injection attempts was used for each data point and their standard deviation as the corresponding error bars. (**b**) Threshold injection line current density for both injection lines as a function of temperatures.

While our simulations showed that a short but strong current pulse can nucleate a domain, experimental injection typically requires a relatively low current density of ~10^11 ^A/m^2^ and pulse width of ~10 ns. The difference is likely to originate from the relatively high temperatures that are generated by the significant Joule heating. To study the effects of temperature, we simulated the pulse duration required for deterministic domain wall injection as a function of current density for the two injection line geometries and at different temperatures as shown in Fig. 6(a). Temporally static and spatially uniform temperature distributions were considered. With the addition of a randomly fluctuating thermal field^32^, *t*_*p*_ for different simulation runs were found to differ. Therefore, the average of 25 injection attempts was used and their standard deviation is shown as the error bars.

As the temperature was increased, *t*_*p*_ and *J*_th_ were found to decrease; i.e. less energy was required for domain injection. Figure 6b shows the injection current density as a function of temperature. The *J*_th_ at 1000 K was found to be about 39% lesser than at 0 K. Comparing these simulation results with experimental observations, *t*_*p*_ was found to be much shorter (~3 ns versus ~10 ns) and *J*_th_ was found to be much larger (~1 × 10^12^ versus ~5 × 10^11^). It is likely that the Joule heating resulting from a current lower than the *J*_th_ will first heat up the nanowire beneath^33^, with magnetization reversal occurring only when a sufficiently high temperature is reached. The proposed injection mechanism not only explains for the low current and high *t*_*p*_ reported in literature but could also play an important role in the energy consumption of domain injection processes.

The numerical calculations in Fig. 6 also show that our design markedly decreases the current required for DW injection. Fitting in the threshold conditions at 300 K, it is calculated that our proposed injection method only consumes 24% of the energy used in conventional injection lines. For the other temperatures, the energy consumption were also calculated to be around 30%, agreeing well with our experimental results. The large energy savings we have presented here will not only path the way to energy efficient magnetic memory devices but also improve data stability by limiting Joule heating in the nanowires.

## Conclusion

A highly angular Π-shaped injection line was proposed and demonstrated to be highly efficient at the injection of data bits into perpendicular magnetic anisotropy magnetic nanowire. The structure of our proposed stripline generates a highly shaped electric current which in turn generates an extremely localized field. Under such intense magnetic fields, a pair of magnetic bubble domains nucleates and quickly grows into a magnetic domain in the nanowire underneath. Experiments show that our method can deterministically write a magnetic data bit using only a 15 ns pulse with a current density of 5.34 × 10^11^ A/m^2^. Both experiments and simulations show that our injection line design requires about 30% of the energy for bit writing as compared to conventional designs. Furthermore, the simple design of our Π-shaped injection ensures its compatibility with current DW device designs, allowing for widespread adoption.

## Methods

### Film deposition

A silicon wafer with a 300-nm-thick SiO_2_ layer was used as a substrate. Co/Ni multilayer was deposited using DC magnetron sputtering deposition technique at room temperature. The stack structure was, from the substrate side, Ta(5 nm)/Pt(5 nm)/[Co(0.25 nm)/Ni(0.5 nm)]_4_/Co(0.25 nm)/Ta(5 nm).

### Device fabrication

The device was fabricated in three processes: first, the 350 nm wide nanowire was patterned by electron beam lithography and Ar ion milling from the Co/Ni multilayer film. Secondly, two Ta(10 nm) hall bars were patterned using electron beam lithography technique followed by resist lift-off. Third, Ta(6 nm)/Cu(94 nm)/Au(24 nm) electrodes as well as the injection line were also fabricated using electron beam lithography technique and lift-off. Argon reverse sputtering was employed before the second and third processes to obtain a better Ohmic contact.

### Electrical measurement

The Hall resistance measurements and domain wall injection were carried out on a Cascade Microtech probe station. A Picosecond 10300B pulse generator was used to inject domain walls by applying pulsed current from *E* to *E*’. The Hall resistance is determined by measuring the voltage with a Keithley 2000 between electrodes *C* and *D* while applying a constant DC current of 50 μA with Keithley 2400 along the nanowire. All measurements were performed at room temperature.

### Simulations

COMSOL Multiphysics was used to accurately model the current density distribution and its resultant Oersted field by solving the following set of equations:


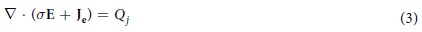



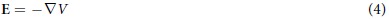



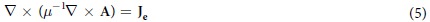



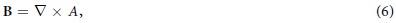



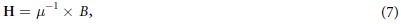


Where *μ* is the permeability, *σ* is the conductivity, *V* is the electric potential, *J*_e_ is the current density, **A** is the magnetic vector potential, **H** is the magnetic field and **B** is the magnetic flux density.

Mumax3 was used to perform the micromagnetic simulation by numerically solving the Landau-Lifshitz-Gilbert (LLG) equation:


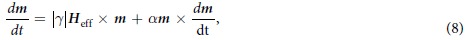


where ***m*** is the unit vector of the local magnetization, *γ* is the gyromagnetic ratio, 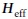 is the effective magnetic field, *α* is the Gilbert damping parameter. The unit cell size is set to 5 nm × 5 nm × 3.25 nm. The material parameters were chosen for Co/Ni:^22^ the anisotropy constant, *K*_u_ = 3.8 × 10^5^ J/m^3^, saturation magnetization *M*_s_ = 6.8 × 10^5^ A/m and the exchange stiffness constant *A* = 1 × 10^−11^ J/m. The damping constant value *α* = 0.02. For the DW injection, the rise time was taken to be 300 ps.

## Additional Information

**How to cite this article**: Zhang, S. F. *et al*. Highly Efficient Domain Walls Injection in Perpendicular Magnetic Anisotropy Nanowire. *Sci. Rep.* **6**, 24804; doi: 10.1038/srep24804 (2016).

## Supplementary Material

Supplementary Information
