# Supplementary material for: Highly Efficient Domain Walls Injection in Perpendicular Magnetic Anisotropy Nanowire
Source: Sci Rep. 2016 Apr 21;6:24804. doi: 10.1038/srep24804 (PMC4838865; doi:10.1038/srep24804)
Supplement: Supplementary Information [file srep24804-s1.doc]

**Highly Efficient Domain Walls Injection in Perpendicular Magnetic Anisotropy Nanowire**

S. F. Zhang^1,2^, W. L. Gan^1^, J. Kwon^1^, F. L. Luo^1^, G. J. Lim^1^, J. B. Wang^2^, W. S. Lew^1*^

*^1^School of Physical and Mathematical Sciences, Nanyang Technological University, 21 Nanyang Link, Singapore 637371*

*^2^Key Laboratory for Magnetism and Magnetic Materials of Ministry of Education, Lanzhou University, Lanzhou, 730000, People’s Republic of China*

**SUPPLEMENTARY INFORMATION**

The influence of defects in the domain nucleation process was studied by introducing magnetic grains onto the magnetic nanowire. This was effected by a voronoi tessellation operation which divided the magnetic nanowire into grains with a mean width of 10 nm, in which each grain varied in magnetic anisotropy energy *K_u_*, anisotropy axis and saturation magnetization by 15%, as shown in Figure S1(a). The exchange coupling between grains was also set at 95%. The injection dynamics at threshold injection current density for the conventional and П-shaped injection line are shown in Figure S1(b) and (c), respectively.

With the introduction of defects, the threshold injection current density did not show any major changes but the pulse duration was observed to decrease. While the defect-free nanowires required 930 and 1350 ps for injection, the presence of defects allowed for domain nucleation in 700 and 900 ps for the conventional and П-shaped injection line respectively. Even at 0 K, the pulse duration for domain nucleation shows a large variation due to the randomly generated grains for each simulation. The presence of defects with low perpendicular magnetic anisotropy served as nucleation points. However, the defects also created pinning sites that increased the threshold magnetic field for field-driven domain propagation and expansion. Both the nucleation of smaller metastable domains and the field-propagation of such domains are critical and their opposite effects in a defect-laden nanowire cancels out, resulting in a negligible change in threshold injection current. Meanwhile, the decrease in pulse duration is explained by the fast nucleation of smaller domains. As shown in Fig S1 (b-c), metastable domains in defect-laden nanowires are formed by 150 ps, while the same occurs in defect-free
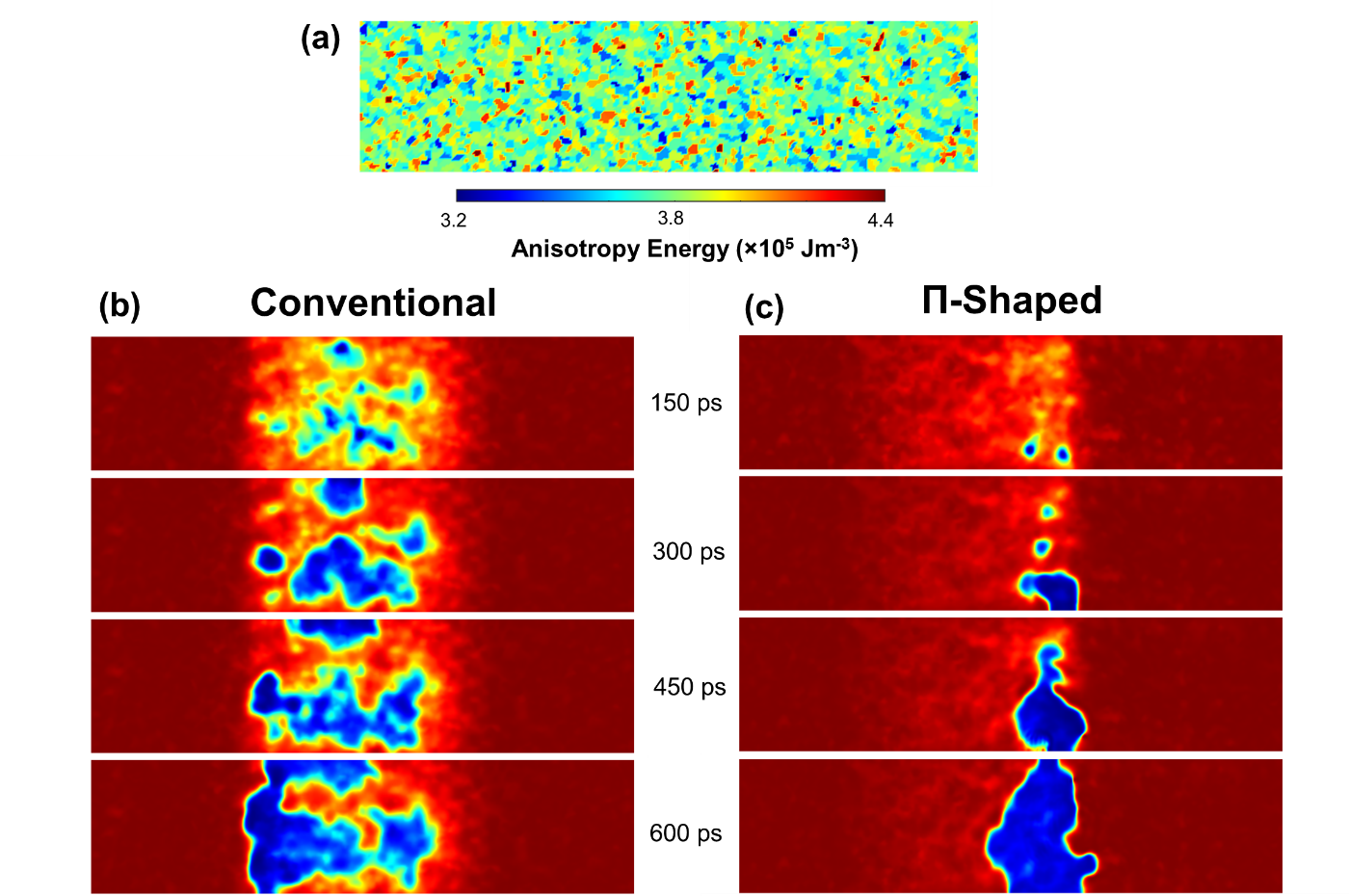
nanowires only at about 400 ps.

Figure S1: (a) Anisotropy energy distribution of a nanowire tessellated with a random seed. (b) Domain injection dynamics at threshold injection current density for the (b) conventional and (c) П-shaped injection line.
